# Unusual coexistence of extramedullary plasmacytoma and nasopharyngeal carcinoma in nasopharynx

**DOI:** 10.1186/s13000-015-0405-y

**Published:** 2015-09-17

**Authors:** Ri-chang Du, Hai-nan Li, Wei Huang, Xiao-ying Tian, Zhi Li

**Affiliations:** Department of Pathology, Yue-bei People’s Hospital, 133, Hui-min Road (South), Shaoguan, 512026 China; Department of Pathology, The First People’s Hospital of Shaoguan City, 3, Dongdi Road (South), Shaoguan, 512000 China; School of Chinese Medicine, Hong Kong Baptist University, 7, Baptist University Road, Kowloon Tong, Hong Kong, China; Department of Pathology, The First Affiliated Hospital, Sun Yat-sen university, 58, Zhongshan Road II, Guangzhou, 510080 China

## Abstract

Nasopharyngeal carcinoma (NPC) is an EBV-associated malignant tumor of nasopharynx. As extremely rare condition, the second primary cancer of nasopharynx can occur in NPC patients synchronously or subsequently. Extramedullary plasmacytoma (EMP) is a rare tumor and commonly originates in the head and neck region. However, there is no report to describe a collision tumor of NPC and EMP occurring in the same nasopharyngeal mass. We report here an unusual case of synchronous coexistence of NPC and EMP occurring in the nasopharynx of an old male patient. A 63-year-old male patient presented with a 3-month history of right-sided nasal obstruction and recently intermittent epistaxis without enlargement of cervical lymph nodes. The solitary mass of nasopharynx was found by radiological and nasopharyngeal examination. Histologically, the mass contained two separated portions and displayed typically histological features of NPC and EMP, respectively. In EMP portion, the tumor was composed of monomorphic plasmacytoid-appearing cells with immuno-positive to CD79a, CD138, CD38, MUM-1 and CD56, but lack immunoreactivity to pan-CK (AE1/AE3), CD20, CD21 and EBERs. In NPC portion, the tumor cells formed irregular-shaped islands with diffusely immuno-positive to pan-CK (AE1/AE3), EMA and EBERs, but lack expressions of lymphoplasmacytic markers. A diagnosis of simultaneous occurrence of EMP and NPC in nasopharynx was made. There was no evidence of tumor recurrence or metastasis 18-month follow-up after radiotherapy. To our knowledge, it may be the first case of coexistence of EMP and NPC synchronously. In addition, the histological differential diagnosis and relevant potential mechanism of this unusual collision tumor were also discussed.

## Background

Extramedullary (extraosseous) plasmacytoma (EMP) is localized plasma cell neoplasm that arises in tissues other than bone. It constitutes 3–5 % of all plasma cell neoplasm [1], and approximately 80 % of EMPs occur in the upper respiratory tract, including the oropharynx, nasopharynx, sinuses and larynx [2, 3], although they have also been described in other rare sites, such as gastrointestinal tract [4], bladder [5], central nervous system [6] and thyroid [7]. EMPs in head and neck are seldom positive for Epstein-Barr virus (EBV) [8], although they usually involve the submucosal lymphoid tissue of the nasopharynx or paranasal sinuses, a region rich in EBV-associated tumors. However, nasopharyngeal carcinoma (NPC) is an EBV-associated malignant tumor that has a high incidence in southern China and Southeast Asia [9]. As exceedingly rare condition, a collision tumor of NPC and EBV-associated lymphoma, such as Hodgkin lymphoma, has been described in the literatures [10, 11]. However, so far there is no report to describe a coexistence of NPC and an EBV-negative EMP in nasopharynx simultaneously in the worldwide. Herein we describe a case of synchronous occurrence of NPC and EMP in nasopharynx at diagnosis. To our best knowledge, it may be the first description of such an event occurring.

**Fig. 1:**
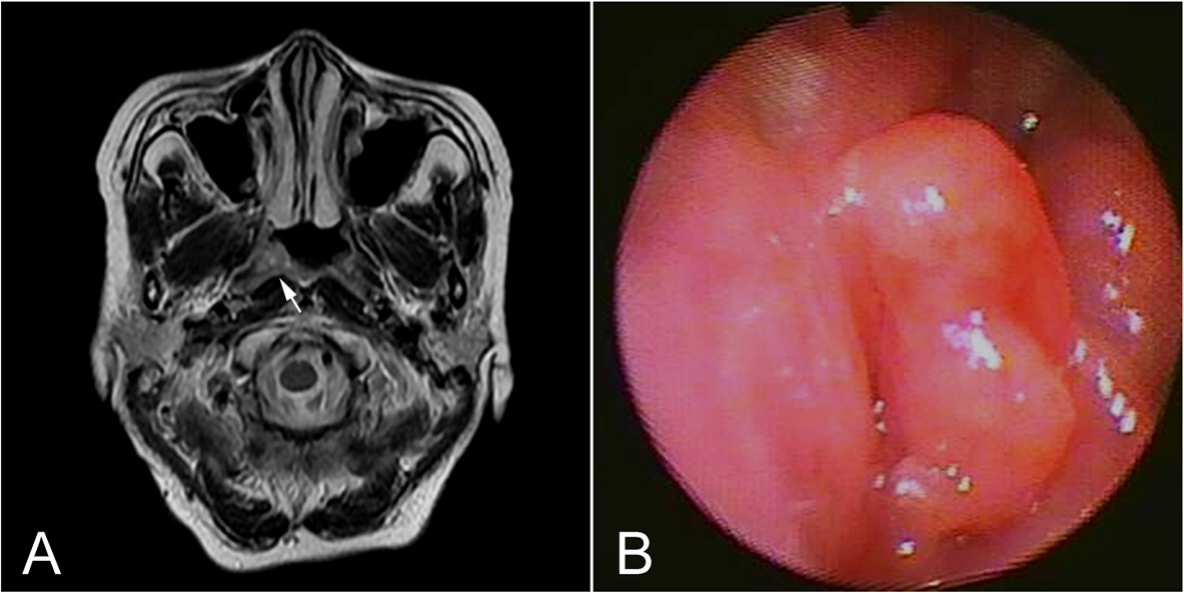
Radiological and nasopharyngeal endoscopic examination. **a**. MRI revealed a mass protruded from the mucosa of nasopharynx (white arrow). **b**. Nasopharyngeal endoscopic examination revealed that a smooth well-circumscribed mass extended from the right fossa of Rossenmuller

**Fig. 2:**
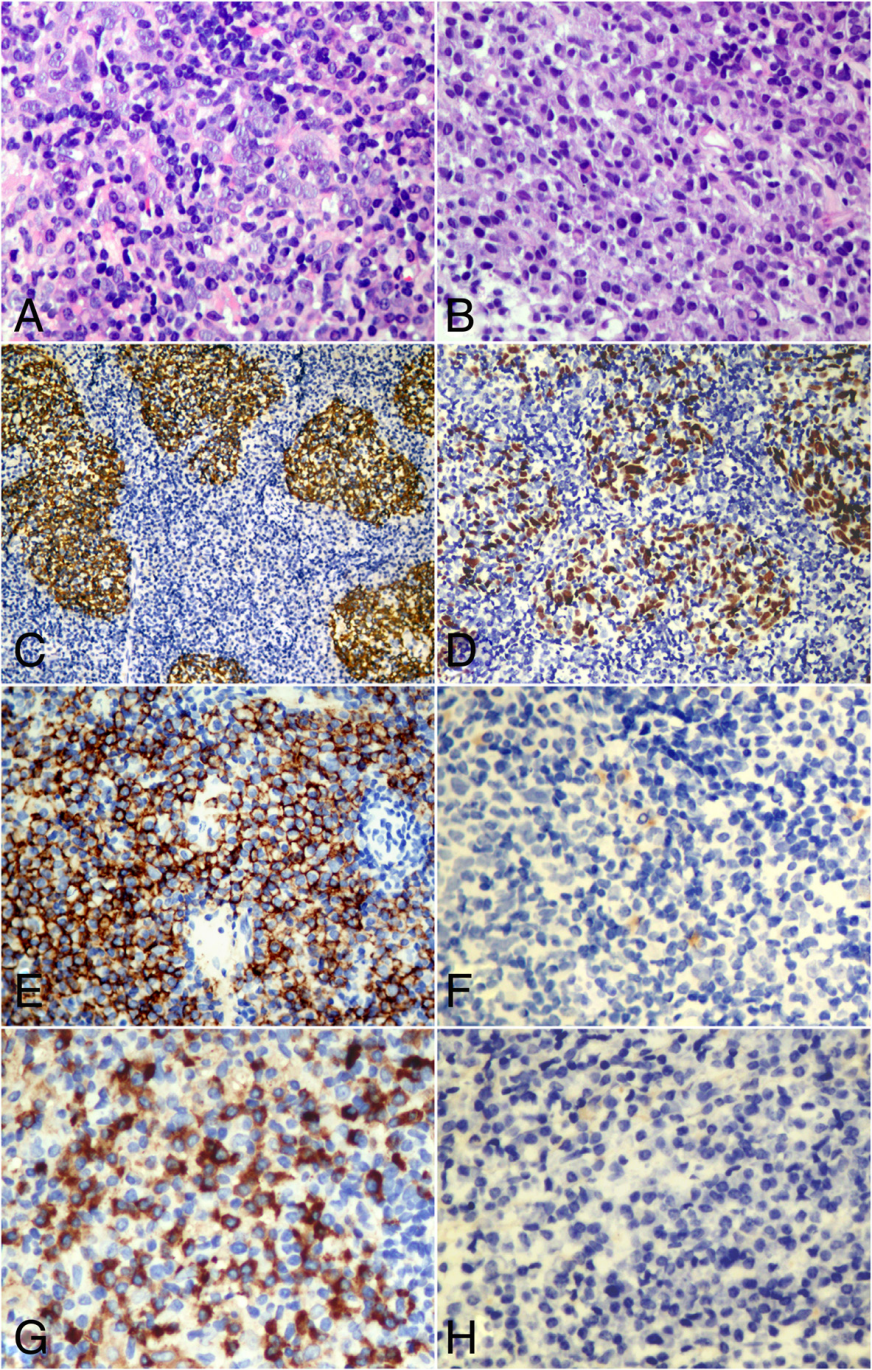
Histological and immunohistological features of the first nasopharyngeal biopsy. **a**. There were several irregular-shaped cell islands found in some areas of tissue. The cells were syncytial appearance with indistinct cell borders, oval vesicular nuclei and large central nucleoli. **b** In other areas, diffuse infiltration of monomorphic plasmacytoid-appearing cells was observed in the subepithelial tissue. **c**. Immunohistochemically, syncytial cell islands were diffusely positive to pan-CK (AE1/AE3). **d**. *In situ* hybridization assay showed that the syncytial cell islands were EBERs-positive. **e**. However, the monomorphic plasmacytoid cells were diffusely immunoreactive to CD138, but lack the expression of CD20 **f**. **g**. The presence of kappa light chain restriction was also detected in plasmacytoid cells. **h**. *In situ* hybridization assay revealed that plasmacytoid cells were EBERs-negative. **a**-**b**, HE staining with original magnification × 400; **c**, **e**-**g**, immunohistochemical staining with original magnification × 400; **d**, **h**, *in situ* hybridization for EBERs with original magnification × 400)

**Fig. 3:**
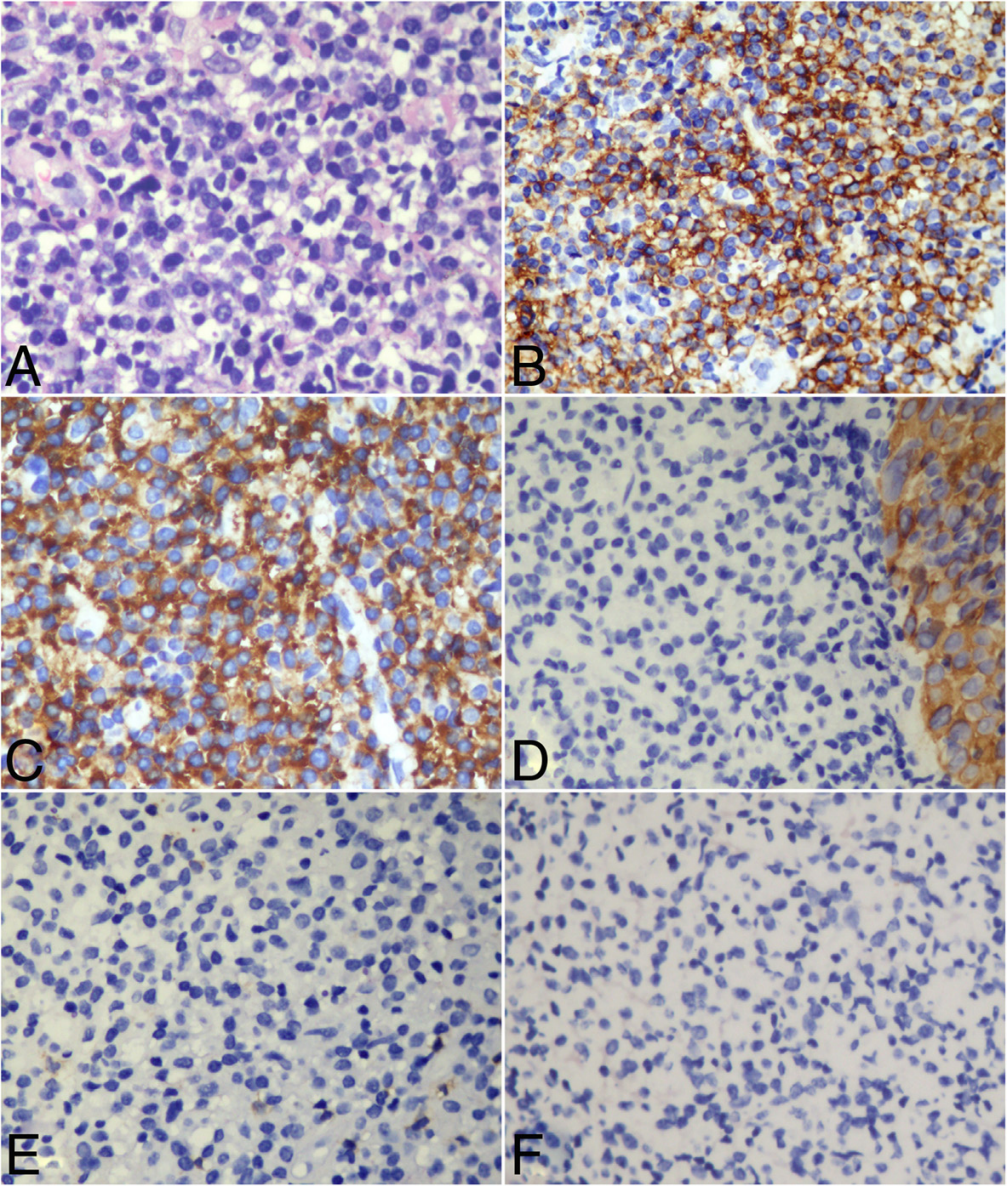
Histological and immunohistological features of the second nasopharyngeal biopsy. **a**. Subepithelial tissue was infiltrated by sheets of a monotonous population consisting of medium-sized atypical plasmacytoid cells without syncytial cell islands. **b**. immunohistochemical staining showed that the plasmacytoid cells were diffusely positive to CD138 and kappa light chain **c**, but they were negative to pan-CK (AE1/AE3) (the neighboring epithelium was positive to pan-CK) **d**, CD20 **e** and not reactive to EBERs detection **f**. **a**, HE staining with original magnification × 400; **b**-**e**, immunohistochemical staining with original magnification × 400; **f**, *in situ* hybridization for EBERs with original magnification × 400)

## Case presentation

### Clinical manifestation and management

A 63-year-old Chinese male patient admitted to our hospital with the complaints of a 3-month history of right-sided nasal obstruction and recently intermittent epistaxis. He had no remarkable medical or family history and physical examination was normal. There were no risk factors of NPC, such as consumption of salt-preserved fish, tobacco smoking, and familiar susceptibility of NPC verified. The neck exam was benign without appreciable lymphadenopathy. Nasopharyngeal examination revealed a smooth mass extending inferiorly from the right fossa of Rossenmuller and effacing the posterior pharyngeal wall. Routine laboratory test results, including blood count, differential, liver and renal function, were within the normal range. Serum and urine assays for M protein and Bence-Jones protein were normal, and there was no evidence of gammopathy. The serologic assessment for titers of immunoglobulin A against EB viral capsid antigen (VCA-IgA) and early antigen (EA-IgA) were positive. Magnetic resonance imaging (MRI) of the nasopharynx showed the mass protruding from nasopharynx (Fig. 1). An endoscopic examination and excisional biopsy was performed. The final histological diagnosis was synchronous occurrence of NPC and EMP in nasopharynx.

After diagnosis, the patient underwent radical radiotherapy with a 45Gy dose in 20 fractions of the nasopharyngeal field, which remarkably reduced the tumor size and relieved nasal obstruction. However, his intermittent epistaxis did not improve. After the radiation therapy, the patient was referred to a whole body F18-fluorodeoxyglucose (FDG) positron emission tomography (PET)/CT study to search for the potentially secondary tumor. But there were no detectable osteolytic lesions and metastatic lesions. Bone marrow biopsy was also performed for excluding multiple myeloma, but the result was normal (Data not shown). The second endoscopic examination and excisional biopsy was performed. Histological examination revealed that only EMP could be observed in the nasopharyngeal tissues. He then received additional radiation therapy (60Gy/30 fractions). The patient was then followed up 18 months after the completion of treatment. Physical examination, MRI of the nasopharynx and neck, and a bone marrow aspiration were performed. There was no evidence of relapse or metastasis.

### Pathological findings

Both excisional biopsies were routinely fixed in 10 % neutral buffered formalin. The tissues were embedded in paraffin. Four micrometer-thick sections were stained with H&E. Immunohistochemical analyses were performed using the ChemMate Envision/HRP Kit (Dako, Glostrup, Denmark). The antibodies used in this study included a broad panel of antibodies against pan-cytokeratin (AE1/AE3), epithelial membrane antigen (EMA), CD20, CD21, CD79α, CD3, CD5, CD138, CD38, MUM-1, CD56, kappa and lambda light chain. The antibodies were obtained from Dako Cytomation (Glostrup, Denmark) and Santa Cruz Biotechnology (Santa Cruz, CA, USA). *In situ* hybridization for EBERs (EBV-encoded RNAs) was performed on the biopsies by EBERs detection kit (Glostrup, Denmark).

Microscopically, the first biopsy showed a diffuse infiltration of monomorphic round-to-oval cells with eccentric nuclei in the subepithelial tissue. These round cells were plasmacytoid-appearing with coarsely clumped chromatin and amphophilic cytoplasm. Mitotic figures were present. However, several irregular islands were also found in the subepithelial tissue. The cell islands were composed of syncytial-appearing large cells with indistinct cell borders, round to oval vesicular nuclei and large central nucleoli. These cells appeared to be crowded and overlapped, and active mitotic figures were present (Fig. 2). Immunohistochemically, the syncytial cell islands were strongly positive to cytokeratin (AE1/AE3) and EMA, but negative to lymphoplasmacytic markers. However, the plasmacytoid cells were positive to CD79a, CD138, CD38, MUM-1 and CD56. The presence of kappa light chain restriction was also detected in plasmacytoid cells. But they were negative to CD20, CD21, CD3, and CD5. EBV detection by *in situ* hybridization for EBERs showed the nuclear positive only in syncytial cell islands, and the plasmacytoid cells were EBV-negative. In the second nasopharyngeal biopsy, subepithelial tissue was infiltrated by sheets of a monotonous population consisting of medium-sized atypical plasmacytoid cells without syncytial cell islands. Immunohistochemically, the tumor cells were strongly positive for CD79a, CD138, CD38 and MUM-1, as well as the presence of kappa light chain restriction. EBV detection was non-reactive (Fig. 3).

On the basis of histopathological features and immunohistochemical phenotypes, its extramedullary location, the presence of EBV-positive syncytial cell islands and light chain restriction in plasmacytoid cells, a diagnosis of synchronous occurrence of NPC and EMP in nasopharynx was made.

## Discussion

Extramedullary plasmacytomas (EMPs) are rare tumors, which represent 3 % of plasma cell neoplasms and commonly originate in the head and neck region [1]. Plasma cell neoplasm has been classified into three subtypes. The most common type is multiple myeloma, which is usually a disseminated disease and is characterized by abnormal M protein. The other two types, solitary plasmacytoma of the bone and EMP of the soft tissue, are considerably less common. Approximately 80 % EMPs occur in head and neck region, and 40 % occur in the nasal cavity and paranasal sinus, 20 % in the nasopharynx, and 18 % in the oropharynx [12]. There is a greater male preponderance (male: female ratio, 3:1) and they occur during the fifth and seventh decades of life [13]. The diagnostic criteria of EMP of the soft tissue have been identified: (a) pathological tissue evidence of monoclonal plasma cells involving a single extramedullary site; (b) no bone marrow involvement; (c) negative skeletal survey results; (d) no anemia, hypercalcemia or renal impairment caused by plasma cell dyscrasia; (e) low serum or urinary levels of monoclonal immunoglobulin [14]. Moreover, EMP can be staged according to the spread of the disease. Stage I is disease confined to one site. Stage II includes tumors with local extension of lymph node involvement. Stage III has metastatic spread. In the present case, the tumor is localized in the nasopharynx and observed to have monoclonal plasma cells proliferation with mildly cellular atypia.

Due to its rarity, the diagnosis of EMP should be made cautiously in clinical practice. The diagnosis is based on a combination of pathological, radiological and clinical features, because this tumor may sometimes be the first manifestation of a systemic disease, multiple myeloma (MM). The reported conversion rate of EMP to MM is 15–20 %, and is associated with a poorer prognosis [1]. However, generalized bone marrow involvement is typically present in MM. Radiological examination reveals osseous lytic lesions, osteoporosis or fractures, which is often associated with bone pain and hypercalcemia. M-protein is found in serum or urine in about 97 % of patients [15]. These radiological and clinical features can not be observed in EMP. Solitary plasmacytomas of the bone (SPB) should also be distinguished from EMP. Unlike EMPs usually presenting as submucosal masses in soft tissues, SPB is a localized bone tumor, and complete skeletal radiographs show no other lesions. There are no clinical features of MM and no evidence of bone marrow plasmacytosis except for the solitary lesion [14, 16]. In our case, there were no clinical features of MM found in patient, PET/CT and bone marrow biopsy showed no abnormalities. There was no evidence of infiltration to the bone marrow or multiple bone destruction at the diagnosis. The clinical features and histopathological findings of our case are consistent with well-differentiated EMP of nasopharynx, Stage I.

For the present case, another important differential diagnostic consideration that needs to be excluded is extranodal marginal zone lymphoma of mucosa-associated lymphoid tissue (MALT lymphoma) in nasopharynx. On the one hand, MALT lymphoma represents one of the commonest hematolymphoid malignancies arising in the salivary glands and head and neck. On the other hand, plasmacytic differentiation is frequently found in MALT, and histological and immunohistochemical features of plasmacytic differentiation in MALT are similar to those of plasmacytoma [17]. The diagnosis of EMP must strictly exclude the MALT lymphoma with prominent plasmacytic differentiation [18]. Based on the criteria defined by WHO classification of tumors of haematopoietic and lymphoid tissues, MALT lymphoma is a lymphoma composed predominantly of small cells with CD20-positive, CD79a-positive and expression of marginal zone cell-associated antigens CD21 and CD35 [17]. In the present case, the complete absence of CD20 and CD21 expression argues against a diagnosis of MALT. In addition, EMP-like post-transplant lymphoproliferative disease (PTLD) has previously been described, and this entity is often associated with EBV [19]. However, the lack of a history of transplant or immunodeficiency and absence of EBV infection in EMP tumor cells rule out this rare disease.

Nasopharyngeal carcinoma (NPC) presents as an epithelial cancer with histology that ranges from keratinizing and non-keratinizing forms. This tumor is endemic in Southern China and Southeast Asia [9]. The undifferentiated variant of NPC is universally associated with EBV. Positive serology against EBV is found in close to 100 % of patients with non-keratinizing NPC, and VCA-IgA and EA-IgA/IgG are the most extensively used diagnostic tool, with positive for NPC varying from 69–93 % [20]. The underlying pathogenesis for EBV-related cancers has not been completely defined, but appears to be mediated through activation of various cell signaling pathways by EBV nuclear antigens (EBNAs) and latent membrane proteins (LMPs). Some studies indicated that different types of latent EBV expression profiles were associated with different malignancies. For example, a type I latency pattern is typical of Burkitt’s lymphoma, and type II is seen in NPC and Hodgkin lymphoma, as well as PTLD [21]. Several cases of NPC and Hodgkin lymphoma occurring in the same patients synchronously or sequentially have been documented in the literature [10, 11]. In those cases, dual EBV infection and different oncogenic pathways activated by the same type of EBV latency profile were suggested for coexistence of NPC and Hodgkin lymphoma [22]. However, the same hypothesis seems not suitable for the current case, because EMP in our case is EBV-negative, although EBV-associated EMPs occurring in head and neck have been described in literature [23]. To the best of our knowledge, our case may be the first case of the simultaneous occurrence of these two tumors.

Recent studies have focused on second primary cancers in NPC patients and indicated that immune suppression, shared genetic factors, and shared environmental risk factors might be the most important reasons to explain the coexistence of NPC and other cancers in nasopharynx [24]. Since cellular immunity is suppressed in patients with NPC and the suppressed condition still remains after remission [25], immune suppression might explain the increased incidence of cancers, especially non-Hodgkin lymphoma, after NPC. There are some common genetic susceptibility involved in NPC and other cancers. For example, polymorphisms in two DNA repair genes, XRCC1 and HOGG1, have been reported to increase the risk of both lung cancer and NPC [26]. Abnormal expression of XRCC1 or HOGG1 was also found in myeloid leukemia and the majority of follicular lymphomas. These genetic abnormalities might explain the coexistence of NPC and lung cancer, myeloid leukemia, as well as follicular lymphoma. However, so far there is no certain shared genetic alteration found both in NPC and EMP. One study has previously reported the presence of RAS mutations only in extramedullary plasma cells and not in intramedullary plasma cells, suggesting a role for RAS mutations in the development of EMP [27]. It is well known that RAS gene abnormalities are closely associated with the development and progression of NPC. We wonder if there are some potential links between RAS mutation and synchronous occurrence of NPC and EMP. The further studies should focus on those abnormal genetic changes, which share in NPC and EMP and promote the development and progression of NPC and EMP simultaneously.

Environmental risk factor is another important factor for tumor development. It has demonstrated that diet habits, particularly Chinese-style salted fish, are common environmental risk factors for tongue cancer, brain tumor and NPC [28]. The environmental factors involved in the simultaneous development of EMP and NPC remain unclear. Since NPC has mainly been treated with radiotherapy, radiation is likely to be an important factor for malignancies occurrence after NPC treatment. However, in the present case, the coexistence of NPC and EMP did not support this hypothesis. We postulate that the EMP in nasopharynx may have evolved as a result of a progression from a polyclonal inflammatory population concomitant to the NPC. Immune suppression and chronic inflammatory stimulation might promote a clonal evolution of plasma cell, leading to a plasmacytoma and collision tumor with NPC. However, this postulation will be required to ascertain in the further studies.

Plasmacytomas are generally associated with a more favorable prognosis compared to MM. In most cases the lesions are eradicated with local radiation therapy. About 70 % of patients remain disease free at 10 years [18]. Our presenting case also showed a favorable prognosis after radiotherapy, and there was no evidence of relapse or metastasis. This indolent course may suggest that many cases of EMP are more closely related to MALT lymphoma than to myeloma. However, subsequent follow-up information of our patient will be required to evaluate the biological behavior of collision tumor of EMP and NPC.

## Conclusion

In conclusion, our presenting case may be the first documented case of coexistence of NPC and EMP in nasopharynx synchronously. Although the precise mechanism involved in synchronous occurrence of two malignancies remains unknown, it should be reflected that there may be a relationship between NPC and EMP. Immune suppression and chronic inflammatory stimulation might play important role in their carcinogenesis. Because of its rarity, there exist diagnostic challenges for pathologists to differentiate EMP in nasopharynx from chronic inflammation or other low-grade B-cell lymphoma with plasmacytic differentiation. It is important to be able recognize the combination of two tumors in same site in order to avoid potential misdiagnosis and improper management of afflicted patients. A longer period of follow-up and more case investigation are necessary to better clarify the biological characteristics and clinical outcomes of this unusual collision tumor.

## Consent

Written informed consent was obtained from the patient for publication of this case report and any accompanying images. A copy of the written consent is available for review by the Editor-in-Chief of this journal.
